# A bilingual systematic review of South Korean medical tourism: a need to rethink policy and priorities for public health?

**DOI:** 10.1186/s12889-021-10642-x

**Published:** 2021-04-06

**Authors:** Qing Xu, Vidya Purushothaman, Raphael E. Cuomo, Tim K. Mackey

**Affiliations:** 1 Department of Healthcare Research and Policy, University of California, San Diego – Extension, San Diego, CA USA; 2 Global Health Policy and Data Institute, San Diego, CA USA; 3 S-3 Research LLC, San Diego, CA USA; 4 Department of Anesthesiology and Division of Infectious Diseases and Global Public Health, University of California, San Diego School of Medicine, 8950 Villa La Jolla Drive, A124, La Jolla, CA 92037 USA

**Keywords:** Medical tourism, South Korea, International health policy, Health policy, Globalization and health

## Abstract

**Background:**

In 2016, the “Act on Support for Overseas Expansion of Healthcare System and Attraction of International Patients” was enacted by the South Korean government in an attempt to accelerate growth of its medical tourism industry. However, only a few years after its implementation, the benefits are not well understood, nor have the positive or negative impacts of expanding Korea’s medical tourism sector been properly evaluated.

**Objective:**

We aimed to systematically review and summarize existing literature describing South Korea’s medical tourism policy and legislative history, while also assessing the impact of this domestic policy approach on the country’s public health systems.

**Methods:**

A bilingual systematic literature review was conducted per PRISMA guidelines for all South Korean medical tourism legislative and policy literature using MeSH terms and other related keywords in two academic databases, PubMed and JSTOR. Published studies were included if they directly addressed South Korean medical tourism policy. To supplement results from the peer-review, the grey literature was also searched using Google search engine for relevant policy documents, information from government websites, and national statistics on medical tourism-related data.

**Results:**

This review included 14 peer-reviewed journal articles and 9 websites. The majority of literature focused on the legislative history of South Korea’s pro-medical tourism policy, economic considerations associated with industry growth, and the specific experiences of medical tourists. There was a lack of studies, analytical or commentary-based, conducting in-depth analysis of the healthcare impact of these policies or comparing benefits and costs compared to other medical tourism destinations. Proponents of medical tourism continue to advocate the government for increased deregulation and investment in the sector.

**Conclusion:**

This systematic review suggests that policy decisions may prioritize economic growth offered by medical tourism over negative effects on the healthcare workforce, access and equity, and its potential to undermine Universal Health Coverage. South Korea continues to examine ways to further amend the Act and grow this sector, but these actions should be taken with caution by critically examining how other countries have adapted their policymaking based on the real-world costs associated with medical tourism.

## Background

Medical tourism is described as the practice of tourists electing to travel across international borders to receive some form of medical treatment in a country outside of their place of primary residence [1]. The main motivations for this movement of millions of patients globally are broadly classified as seeking better-qualitycare, lower cost of services, and quicker access, all factors that are impacted by macro policy issues such as the individual freedom of persons to seek treatment overseas, lack of binding legal frameworks on medical tourism, cross-border arrangements (such as immigration and trade agreements), and patient ethical and privacy issues [2–5]. Estimating the market size and scope of this specific sector of the international healthcare industry can be challenging as there is generally no universally agreed upon definition of what constitutes medical tourism or travel and there is also a lack of verifiable data at the national level due to each government’s reluctance to share information due to market competition concerns [2, 3].

Allied Market Research, a firm that the South Korean government cited in its official reports, forecasted that the global market for medical tourism was projected to be worth $143.8 billion by 2022, representing compounded annual growth of 15.7% from 2015 to 2022 [6]. These estimates are based on projections that include patients traveling across international borders for both critical and less critical but still necessary procedures in addition to elective out-patient single day procedures (e.g. aesthetic, cosmetic and dental treatment) [6]. Though verifiable data about medical tourism may be limited, several sources indicate that the main destinations for medical tourism are located in Asia [2, 7]. These include “Key Destination” countries of Thailand, India, Singapore, Malaysia, and the Philippines, with South Korea being classified as an “Emerging Destination.” [7–12] Collectively, these rival regional economies set the stage for South Korea’s own domestic approach to medical tourism policy reform that will be described and explored in this review [13].

In recent years, due to the government's support, South Korea is becoming one of the top 10 destinations for medical tourism globally, with a focus on promoting its high-quality clinical medicine, technical proficiency of its physicians, and cutting-edge medical technology [14–17]. This emergence was catalyzed in 2016 when the South Korean government launched the “1^st^ Comprehensive Plan to Support Overseas Expansion of the Healthcare System and Attraction of International Patients” (the “Comprehensive Plan”). The plan represented the policy implementation phase of Article 18 of the “Act on Support for Overseas Expansion of Healthcare System and Attraction of International Patients” (“the Act”), a piece of national legislation that was passed in July 2016. Together, the Act and the Comprehensive Plan are the result of concerted efforts by the South Korean government to promote and globalize its domestic healthcare industry by intensifying its competition in attracting foreign patients for the global medical tourism market, which is considered a high value-added industry.

The Act was clear about its legislative intent: expansion of domestic medical services for overseas patients; enabling foreign patient access, appeal, and convenience to safe and high-quality South Korean medical services; and using revenue from medical services offered to foreigners as a vehicle for national economic growth. Two years after the Act, in May 2018, the government launched the “Enforcement Plan to Support Overseas Expansion of Healthcare System and Attraction of International Patients 2018” (“the Enforcement Plan”). Specifically, the South Korean government enacted the Enforcement Plan to adjust and reset the goals of its domestic medical tourism industry push after conducting additional economic and policy assessments. Hence, the progression from legislative action through the Act, implementation through the Comprehensive Plan, and monitoring and adjustment via the Enforcement Plan, represents an important policy experiment to examine in the context of global medical tourism trends and implications for domestic and international health policy.

Despite optimistic economic growth projections, there is local opposition to the South Korean government’s efforts to promote its medical tourism industry. The Korean Federation of Medical Activists Groups for Health Rights (KFHR), an organization that promotes human rights, the right to health, and the rights of healthcare workers (also supported by the Association of Physicians for Humanism, Association of Korea Doctors for Health Rights, Korea Dentists Association for Health Society, and Korean Pharmacists for Democratic Society and Solidarity for Worker’s Health) has argued that investing in medical tourism could lead to commercialization of the national public health system. This could lead to increased costs due to expansion of the private health sector and lead to an internal “brain drain” of healthcare professionals (i.e. when domestic healthcare workers leave rural areas to practice in areas with more coverage and higher income potential or when they leave public practice for private practice) to the more lucrative medical tourism market.

National debate and disagreement among stakeholders about the utility of this domestic public policy stance and investment in medical tourism continues. However, there is limited literature discussing medical tourism policy specific to the experience of South Korea, despite the fact that the country offers an important case study for this topic. Hence, the objective of this systematic review is to summarize the different economic and public health impacts of South Korea’s pro policy stance on supporting its medical tourism sector. In addition, the review also provides critical analysis of South Korea’s medical tourism industry compared to other Asian destination countries including Thailand, India, and Singapore, which the South Korean government considers as regional rivals. Based on this comparative analysis, we conclude with a set of recommendations about what the future should look like for South Korea’s medical tourism policy stance.

## Methods

### Search strategy

This systematic review was carried out in accordance with the Preferred Reporting Items for Systematic reviews and Meta-Analyses (PRISMA) guidelines [18], and was conducted in January 2021. The databases PubMed and JSTOR were searched for articles published in English and Korean languages published before January 18, 2021, using the Medical Subject Headings (MeSH) unique ID term “Medical Tourism” (ID: D057193) with “Korea” (ID: D007723). Other non-MeSH key search terms included “Tourism, Medical”, “Health Tourism”, “Tourism, Health”, “Surgical Tourism”, “Tourism, Surgical”, “Medical Tourists”, “Medical Tourist”, “Tourist, Medical”, and “Tourists, Medical” in combination with “Korea” or “Korean”. Additionally, we examined the grey literature using the Google search engine in both English and Korean language to supplement the scientific literature and carry out policy analysis of primary documents available online. The same keywords in the literature review were used in structured web search queries with the addition of the equivalent Korean language terms: 의료관광, 관광 정책, 국제 의료관광 코디네이터, 의료관광마케팅. In order to better understand the policy rationale for South Korea’s activities in promoting its medical tourism industry, we also conducted a descriptive assessment of the policies of other top Asian medical tourism destinations in our grey literature review. In addition to Google keyword searches, we also reviewed and examined documents from select South Korean government websites (Ministry of Health and Welfare, Korea Tourism Organization), which contained information specific to the legislative process and policy language of the Act, the Plan, and the Enforcement Plan. All of these sources were included on the basis of being primary documents or information sources having direct relevance to recent South Korean public policy decisions on medical tourism.

### Inclusion criteria

Articles were included for purposes of analysis in this review if they discussed or analyzed South Korean medical tourism policy, including in comparison or in contrast to other major Asian medical tourism destinations. Major themes detected included analysis in relation to barriers to policy implementation, challenges regarding the operational status of medical tourism policy, barriers to delivering service in the medical tourism sector (e.g., cultural and social background differences), client motivation (e.g., loyalty) to participate in the medical tourism industry, and any assessment of the impact of medical tourism policy (e.g., internal benefit, economic impact) on South Korea. Research articles, review articles, case studies, commentaries and letters to the editor were included for review in this study.

### Exclusion criteria

Studies were excluded if they were conducted or discussed medical tourism policy with a focus on other countries (e.g., Japan, the United States of America, Mongolia, etc.), articles that discussed other important medical issues in South Korea (e.g., HIV, Middle East respiratory syndrome, etc.) but did not focus on medical tourism, articles that focused on immigrant health issues in other destination countries (e.g., the health of Korean Americans in other countries), and articles focused on other medical problems related to globalization of healthcare (e.g., transnational organ transplant industry, stem cell tourism, etc.) We did not exclude literature on the basis of specific content types or study design.

### Study selection

All studies were extracted and assessed for eligibility by QX and VP independently to minimize errors and to reduce potential biases. Discrepancies regarding study eligibility were resolved through discussion among all authors for final consensus.

### Bias assessment

The Cochrane Collaboration risk of bias (RoB) tool was used to assess bias for any randomized controlled trials (RCTs) selected [19]. If comparable quantitative measures were uncovered across studies, the influence of publication bias was to be assessed using a funnel plot. For other types of studies, we qualitatively evaluated the influence of biases which have been previously cited as being influential in health policy research.

## Results

### Search results

**Fig. 1:**
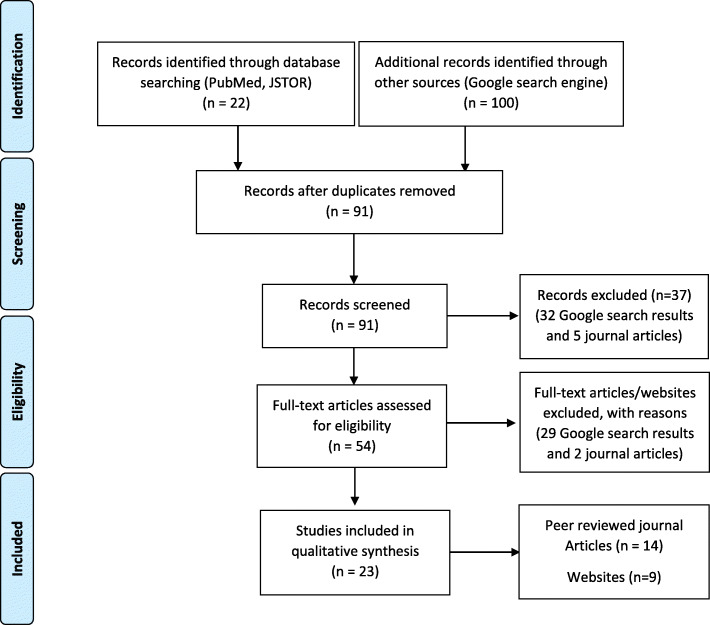
Flowchart of study selection

Based on our review and search criteria, we reviewed a total of 123 results (22 journal articles and 100 Google search results) (see Fig. 1). After excluding one duplicate, 21 published articles were returned on PubMed and JSTOR that were then reviewed for relevance to the study inclusion and exclusion criteria. After review, seven articles were excluded based on a review of the focus of the paper after reviewing the abstract and then reviewing the full text. The major themes identified in the literature focused on: (a) formal policy reviews of South Korea’s medical tourism industry [20]; (b) analysis of current barriers, challenges and successes experienced by the South Korean medical tourism industry [21–25]; (c) proposals to assess the performance of the South Korean medical tourism industry [26]; (d) assessment of utilizing the healthcare worker education system to support further development of the medical tourism industry [27]; (e) examining the relationship between employee satisfaction and performance of medical tourism facilitators [28]; (f) analysis of the effects of price and health consciousness and satisfaction associated with the South Korean medical tourism experience [29, 30]; and (g) factors associated with why certain patients choose to participate in medical tourism in South Korea [31–33].

Based on our Google online search methodology, we reviewed the first 100 results returned (50 in Korean, 50 in English). After removing duplicate results, we retrieved 32 unique English and 38 Korean websites that were then reviewed for relevance and inclusion in this study. Website results in English included 2 government websites, 5 news articles, 2 online articles, and 23 advertisement websites; Korean websites included 2 government websites, 18 news articles, 9 online articles, and 9 advertisement websites. After excluding the 32 advertisement websites that where were not relevant to the study, 38 websites were reviewed for full content and 9 websites (English 7, Korean 2) were identified that directly related to the South Korean medical tourism industry, including proving reports and updates associated domestic medical tourism policy and relevant data. Our grey literature review provided additional details on the specific motivation, rationale, and strategy of the South Korean government in pursuing its medical tourism policy that was not available in the literature. Overall, 14 journal articles (see Table 1) and 9 websites (see Table 2) were eligible for inclusion in the study.

**Table 1:** Studies of Korean medical tourism

	Reference	Article Title	Journal	Content type	Resource	Key Words	Key Findings
1	Choi Wa et al., 2018 [33]	Word-of-mouth in medical tourism: the major determinant for Emirati patients to visit Korea	The Korean Journal of Internal Medicine	Letter to the Editor	PubMed	Medical tourism; Emirati patients; Word-of mouth	There was a perspective gap on medical tourism between Emirati patients and Korean health professionals.
2	Dang HS et al., 2020 [26]	Grey System Theory in the Study of Medical Tourism Industry and Its Economic Impact.	International Journal of Environmental Research and Public Health	Research Article	PubMed	Asia-Pacific region; Grey system theory; Taiwan; economic impact; medical tourism industry; performance evaluation; sustainable development.	Tourism sources and healthcare medical infrastructures play a crucial role in promoting the healthcare travel industry, while cost advantage and marketing effectiveness were less considered.
3	Jun J, Oh KM, 2015 [30]	Framing risks and benefits of medical tourism: a content analysis of medical tourism coverage in Korean American community newspapers.	Journal of health Communication	Research Article	PubMed		Korean American community newspapers are rarely engaged in risk communication and lack sufficient information about potential risks of medical tourism while emphasizing diverse benefits.
4	Kim C et al., 2020 [27]	Is Korea Ready to Be a Key Player in the Medical Tourism Industry? An English Education Perspective.	Iranian Journal of Public Health	Original Article	PubMed	Medical tourism; English education; Nursing; Korea	Nursing major students were satisfied with the English instructors but were not feeling that is enough to deal with foreign patients. Students felt that the teaching methodology should be changed to incorporate more medical content into the nursing English program.
5	Kim KL, Seo BR, 2019 [23]	Developmental Strategies of the Promotion Policies in Medical Tourism Industry in South Korea: A 10-Year Study (2009–2018).	Iranian Journal of Public Health	Original Article	PubMed	Medical tourism, Medical tourism industry, Medical service	International affairs and cultural aspects have a significant impact on the selection of medical tourism.
6	Kim M et al., 2017 [32]	From Servicescape to Loyalty in the Medical Tourism Industry: A Medical Clinic’s Service Perspective.	INQUIRY	Original Article	PubMed/ JSTOR	Republic of Korea; emotion; international medical tourist; loyalty; marketing; medical clinic; medical tourism; servicescapes; structural equation modeling.	The interrelationship of servicescapes, positive emotion, and satisfaction is essential in influencing international medical tourists’ loyalty to a medical clinic.
7	Kim S et al., 2019 [22]	Critical Success Factors of Medical Tourism: The Case of South Korea.	International Journal of Environmental Research and Public Health	Original Article	PubMed	Medical tourism; South Korea; Success factors; Supplier perspectives.	The medical tourism industry not only includes medical services but also involves tourism perspectives, supporting the patient and their companions to stay in a comfortable and pleasurable environment.
8	Park J, et al.,2017 [29]	The Effects of Price and Health Consciousness and Satisfaction on the Medical Tourism Experience.	Journal of Healthcare Management	Review Article	PubMed		Medical tourists’ price consciousness was significant with respect to their satisfaction with medical and travel services. Health consciousness also influenced their decision-making process. Health consciousness did not have a significant effect on tourists’ satisfaction with medical travel services.
9	Park JK et al., 2020 [28]	Exploring Internal Benefits of Medical Tourism Facilitators’ Satisfaction: Customer Orientation, Job Satisfaction, and Work Performance.	Journal of Healthcare Management	Research Article	PubMed		Satisfaction with management is positively correlated with customer orientation and job satisfaction of medical tourism facilitators. Satisfaction with coworkers has a direct impact on customer orientation.
10	Rokni L et al., 2017 [24]	Barriers of Developing Medical Tourism in a Destination: A Case of South Korea.	Iranian Journal of Public Health	Original Article	PubMed	Cultural competence; International healthcare; Korea; Linguistic proficiency; Medical tourism.	Lack of specialty and expertise among the health care practitioners in the scope of cross-cultural communication, seems to be the core barrier to development of medical tourism in Korea.
11	Rokni L et al., 2019 [21]	Improving Medical Tourism Services through Human Behaviour and Cultural Competence.	Iranian Journal of Public Health	Original Article	PubMed	Cultural competence; Human behaviour; Medical tourism; South Korea.	Challenges due to cultural and social background differences: 1) The personal characteristics of doctors. 2) External supports to be provided by the associated organizations. 3) Skillfulness, which implies the culturally oriented interaction with foreign patients.
12	Seo BR, Park SH, 2018 [20]	Policies to Promote Medical Tourism in Korea: A Narrative Review.	Iranian Journal of Public Health	Review Article	PubMed	International meditour coordinator; Korea; Medical sector; Medical tourism industry	In Korea, International Meditour Coordinator (IMC) s are contributing to the development and enhancement of competitiveness in Korea’s global healthcare industry. It is urgently necessary to establish human resource management policy guidelines in the medical tourism industry
13	Shin J et al., 2018 [31]	Utilization Status and Satisfaction with Medical Services in Nonresidential Foreign Medical Tourists Visiting a Korean Medicine Hospital.	Evidence-Based Complementary and Alternative Medicine	Research Article	PubMed		The most frequently used visiting channels were agencies. Nonresidential foreigners who received integrative medicine treatment expressed high satisfaction, but visiting and promotion channels were shown to be limited
14	Sung S, Park HA, 2019 [25]	Perceived cultural differences in healthcare for foreign patients visiting South Korea: tool development and measurement.	BMC Health Services Research	Research Article	PubMed	Cultural differences; Culturally competent healthcare; Medical tourism; Nursing care; Tool development.	Foreign patients visiting South Korean hospitals perceived that the healthcare culture differed significantly from that of their home country.

**Table 2:** Websites related to Korean medical tourism

	Title	Content type	Issuing Body	Explanation	URL
1	Act on Support for Overseas Expansion of Healthcare System and Attraction of International Patients 의료 해외진출 및 외국인환자 유치 지원에 관한 법률”	Act	National Assembly	Language of the specific Law enacted by Korean for promotion of medical tourism	http://www.law.go.kr/%EB%B2%95%EB%A0%B9/%EC%9D%98%EB%A3%8C%ED%95%B4%EC%99%B8%EC%A7%84%EC%B6%9C%EB%B0%8F%EC%99%B8%EA%B5%AD%EC%9D%B8%ED%99%98%EC%9E%90%EC%9C%A0%EC%B9%98%EC%A7%80%EC%9B%90%EC%97%90%EA%B4%80%ED%95%9C%EB%B2%95%EB%A5%A0/(13599,20151222)
2	Analysis of the status of eco-system in the medical tourism industry in Korea and activation of medical tourism (2016) 한국 의료관광 산업 태계 현황분석 및 의료관광 활성화 중장기 전략 수립 용역 실태조사 결과 보고서 (2016)	Report	Korea Tourism Organization	Analysis of the status of eco-system in the Korean medical tourism industry	https://kto.visitkorea.or.kr/file/download/bd/95fefa55-8241-11e7-975f-1fb2990367af.pdf.kto
3	Establish a KSA marketing strategy and action plan for revitalizing medical tourism (2014) KSA 중증환자向의료관광 유치 활성화를 위한 마케팅 전략 및 실행계획 수립(2014)	Report	Korea Medical Holdings	An marketing strategy and action plan proposed by Korea Medical Holdins	http://kto.visitkorea.or.kr/kor/notice/data/report/org/board/view.kto?id=424734&rnum=2
4	Korean Medical Tourism Overview (2013) 한국의료관광총람(2013)	Report	Korea Tourism Organization & Ministry of Culture, Sports and Tourism	An annual medical tourism report commissioned by the Korea Tourism organization & Ministry of Culture, Sports and Tourism	https://kto.visitkorea.or.kr/kor/notice/data/report/org/board/view.kto?id=420214&instanceId=127
5	Korea Medical Tourism Marketing (2016) 한국의료관광마케팅 (2016)	Report	Korea Tourism Organization & Ministry of Culture, Sports and Tourism	Provides an overview of Korean medical tourism marketing	https://kto.visitkorea.or.kr/kor/notice/data/report/org/board/view.kto?id=427142&instanceId=127
6	Korea statistical information service	Database	Statistics Korea	Number of hospitals by Region/ Number of Health center Branch, Healthcare center (1997–2017)	http://kosis.kr/eng/
7	Medical Tourism Promotion Marketing Current and Strategy (2012) 의료관광 홍보 마케팅 현황 및 전략(2012)	Report	Korea Tourism Organization	This study is to establish the basis for the medical tourism development strategy by analyzing the status and characteristics of foreign medical tourists in Korea	https://kto.visitkorea.or.kr/kor/notice/data/report/org/board/view.kto?id=421414&rnum=72
8	Study of developing Cluster Medical Tourist Model (2014) 채류형 의료관광 크러스터 모델 개발연구 (2014)	Report	Ministry of Culture, Sports and Tourism	In order to effectively promote the resident medical tourism cluster project, which is a national government task, this report set up the type and model of medical tourism cluster that can efficiently connect and utilize medical and tourism resources.	https://kto.visitkorea.or.kr/kor/notice/data/report/org/board/view.kto?id=421406&isNotice=false&instanceId=127&rnum=90
9	The awareness of Korean medical Tourism among foreigners (2017) 한국 의료 웰니스 해외인지도조사보고서 (2017)	Report	Korea Tourism Organization & KHADI한국보건산업진흥원	A report to identify needs of foreign medical tourists	https://kto.visitkorea.or.kr/kor/notice/data/report/org/board/view.kto?id=430316&isNotice=false&instanceId=127&rnum=3

In the subsequent sections we provide an overview of how these different sources of information relate to the history of medical tourism legislation and policymaking in South Korea, the rise of the medical tourism industry and data related to its performance, criticism and challenges associated with the industry, and its potential future trajectory.

### Risk of bias

Our review did not uncover RCTs for bias assessment using Cochrane Collaboration guidelines. Prior publications have suggested that publication bias [34], surveillance bias [35], context bias [36], and omission bias [37] may be relevant to health policy research. Quantitative measures uncovered by this systematic review were not comparable across studies, among the small subset of studies where quantitative measures were reported. Therefore, it was not possible to engage in conventional techniques used to assess for these biases. Nevertheless, as consistent with prior literature, it appears valuable to conceptually make room for the possibility that these biases may have affected the overarching findings of this review as follows: (1) there may have been unpublished reports describing lack of economic and public health impacts from medical tourism in South Korea; (2) impacted economic and public health areas may have been more heavily surveilled than non-impacted areas; (3) the threshold for economic and public health impact may be more sensitive for researchers assessing medical tourism in a South Korean setting; and (4) relatively egregious acts of commission by the medical tourism sector in South Korea may be favored for reporting over acts of omission [20].

### Overview of history of South Korea medical tourism policy

The South Korean government’s policy of actively promoting medical tourism began in earnest following the 2009 revision of ‘Medical Service Act’, which opened the door for domestic healthcare entities and other private companies (with the exception of companies in the healthcare insurance industry) to lawfully attract foreign patients with healthcare services [24] (see Table 3 for policy timeline). In conjunction with the ‘Medical Service Act’, in January 2009, the South Korean government announced the “New Growth Engine: Vision and Strategy” plan. According to this strategy, global health care was viewed as one of 17 new growth industries under the category of 5 high value-added industries that the government would strategically explore.

**Table 3:** Timeline of the Korean Government’s Policy Actions in Support of Medical Tourism

Year	Description
Nov 2005	Korean government formed a task force with the objective of attraction of foreign patients
Jan 2009	• Revised the medical law: introducing, arranging, and inviting foreign patients became legal • Attracting foreign patients was selected as a specific task • Designating the Global health care business as one of 17 new growth engine industries. • Registration Act for Foreign Patient Registration (Article 27–2 of the Medical Law)” was passed
May 2009	Implementing registration system of medical institutions Issued medical visa (M visa) for medical tourists
Nov 2009	Developing “Smart Korea, Medical Korea” as a national medical brand
July 2011	Developing malpractice insurance policy for a medical institution
Dec 2011	Allowing physicians and dentists to dispense medicines
May 2013	Designating medical tourism industry as one of 140 national tasks
Nov 2013	Establishing medical tourism hotel business
Sep 2014	Allowing hospital facility to enter medical tourism hotels
July, 2016	“Act on Support for Overseas Expansion of Healthcare System and Attraction of International Patients” enacted
March 2017	“1st Comprehensive Plan to Support Overseas Expansion of the Healthcare System and Attraction of International Patients”
May 2018	“Enforcement plan to Support Overseas Expansion of Healthcare System and Attraction of International Patients 2018”

Source: Statistics on International Patients in Korea (KHIDI 2018)

In February 2013, 140 national priorities were announced by former president Park Geun-hye, with global healthcare being named one of them [38]. This led to the passage of the Act in 2016, but even before its enactment, the South Korean administration implemented a series of policy mechanisms aimed at promoting medical tourism based on separate comparative assessments of the medical tourism industry of other countries. Specifically, the South Korean government analyzed the policy and economic strategies of Thailand, Singapore and India, major competitor countries in the sector, and reported findings in its 2011 Economic Policy Coordination Committee report titled “Performance of and Promotion Measures for Medical Tourism Industry” [39–41]. The results of these evaluations are reflected in policy decisions pre- and post-the Act that will be discussed [26].

For example, one policy mechanism the report identified as a means to encourage medical tourism included the creation of a medical visa system to enable foreign patients’ easier entry and access to the South Korean healthcare system. Other policy changes included giving permission to allow medical advertisements in foreign languages, representing a clear departure from strict regulations on marketing within its own national health care system [42]. In July 2011, in order to make it easier for foreign patients to take action against potential medical errors and to reduce financial risk for foreign patients, the Korea Health Industry Development Institute (KHIDI) developed medical malpractice insurance policy covering domestic and foreign patients [42]. In December 2011, through the revision of Presidential decree of Pharmaceutical Affairs Act, the Ministry of Health and Welfare (MOHW) also allowed physicians and dentists to dispense medicines to foreign patients, despite strict separation for similar diagnosis and dispensing in its public health system.

Further, in order to enable better provision of healthcare services for medical tourists, a new type of business entity called the “medical tourism hotel business” was created with the enactment of a Presidential decree through the ‘Tourism Promotion Act’ on November 2013. The decree established medical hotels as entities that provide services and products similar to what a hotel would offer (e.g. different types of room accommodations, food and beverage services, etc.), while also allowing them to provide various types of medical services in the role of a healthcare facility [43–45]. Additionally, this policy required that South Korean medical tourism hotels only be registered by medical providers or medical tourism business agencies and also developed an International Meditour Coordinator (IMC) to further promote these services within the public health system [20]. Medical tourism facilitators also focused on improving the satisfaction among those working in the medical tourism industry in order to improve service performance and appeal [28].

Following these early minor policy actions to promote medical tourism, in 2016, the Act was signed into law as a response to industry criticism that there was still insufficient legal and institutional support to grow the medical tourism industry [46]. The main legislative elements of the Act were designed to address what was perceived as continued policy shortcomings that inhibited growth of the sector, particularly in the context of growth experienced by other regional destination countries (for a summary see Table 4). Collectively, these provisions established certain criteria and formal evaluation of South Korean medical tourism providers, put in place restrictions on who could provision medical tourism services, created mechanism for foreign patient’s rights and protections, and also enabled broader direct-to-consumer marketing of medical tourism services.

**Table 4:** The main legislative elements of the Act on Support for Overseas Expansion of Healthcare System and Attraction of International Patients (2016)

Section of Act	Content
Art 6	A requirement that a medical institution and a foreign patient attraction agency should have at least one medical specialist assigned for each medical department and that there be mandatory purchase of medical malpractice liability insurance
Art 8	A medical institution and a foreign patient attraction agency should affix the certificate of registration and circulations of patients’ rights and interests
Art 9	Placed a restriction on excessive fees
Art 10	Places a restriction on attraction of foreign patients by a general hospital
Art 14	Required evaluation and designation of a medical institution and an attraction agency
Art 15	Provided permission for medical advertisement in a foreign language

Reflecting a bigger push by the South Korean government to officially promote its domestic medical tourism industry, the entire industrial sector was promoted via a dedicated government sponsored website called “**VISIT MEDICAL KOREA**” (http://english.visitmedicalkorea.com/english/pt/index.do) operated by the Korea Tourism Organization and KHIDI. The website was specifically designed to introduce and promote services from South Korean hospitals and medical facilities to prospective foreign patients and has been translated into 5 different languages including English, Japanese, Chinese, Russian and Arabic. It should be noted that there is no Korean language version for this website.

### Economic considerations of South Korean medical tourism

In 2016, based on legislative goals set by the Act, the Comprehensive Plan projected a substantial increase in the number of foreign patients that would use South Korean medical services from 0.3 million in 2015 to 0.8 million in 2021, with related revenues increasing from 666.4 billion won ($0.55 billion) to 1700 billion won ($1.40 billion) [47]. It also estimated additional added value through the creation of 80,000 jobs and other related medical tourism economic stimulus of 4000 billion won ($3.30 billion) through 2017–2021 [47]. Prior to the passage of the Act, the number of foreign patients visiting South Korea increased from 60,201 in 2009 to 296,889 in 2015, with an annual growth rate of 30.5%, reflecting significant and promising growth [23, 38], which also benefited from the international popularity of Hallyu (Korean pop culture) [22]. In 2015, the distribution of foreign patients by nationality was Chinese (34%), US nationals (14%), Russians (7%), and Japanese (6%). The breakdown of healthcare utilization by medical department as used by medical tourists included internal medicine (21.3%), plastic surgery (11%), health checkup centers (9%), dermatology (9%), and orthopedics (6%). A total of 2,813 South Korean medical institutions attracted foreign patients in 2015 [38].

However, only two years after the Act, the adjusted plan (i.e., the Enforcement Plan) retargeted the projected goals by substantially lowering initial estimates anticipating an influx of new foreign patients. This revision reflected recognition of the need to correct optimistic projections of how fast the medical tourism industry would grow based on the impact of the Act and related pro-medical tourism policies (see summary in Table 5). Following these adjustments, the number of new foreign patients taking part in South Korea’s healthcare system has continued to fluctuate. For example, according to data from the South Korea MOHW, after the passage of the Act in 2016, the inbound number of international patients increased to 364,189, and the total revenue from international patient care grew to 860.6 billion won, which represented an annual average growth of 48.2% [48]. However, in 2017 the reported medical tourists dropped to 321,574, corresponding to a revenue drop of 26% from 2016 [49]. In 2018, the number of medical tourists had again increased to 378,967, reflecting a more modest 17.8% increase from 2017 [50].

**Table 5:** Data from the Comprehensive plan (2016) compared to the Enforcement Plan (2018) - Target Number of Foreign Patients

Year	2017	2018	2019	2020	2021	2022
Plan						
The Comprehensive Plan (2016)	470,000	/	650,000	/	800,000	/
The Enforcement Plan (2018)	/	360,000	400,000	460,000	520,000	600,000

Despite data generally pointing to economic growth of the South Korean medical tourism industry, the 2016 Comprehensive Plan highlighted some key challenges for the sector moving forward, including low overseas awareness of South Korean medical care [33], lack of professional personnel and expertise in attracting foreign patients, cultural differences with foreign patients that impacted service appeal [21, 25], lack of customized medical services for foreign patients, and declining credibility of services due to cases of illegal and unsanctioned activities [24]. Hence, based upon data available prior to and post enactment of the Act, the industry has experienced growth, but officials continue to identify barriers that impede further development of the sector leading to calls for increased investment. There also remain uncertainties about the utilization of education systems that are needed to train a new cadre of healthcare professionals to support the growing workforce needs of the medical tourism industry [27]. Additionally, the potential negative impacts of investing in medical tourism did not appear to be factored into the government’s analysis.

Despite challenges with growth, the national government continued its aggressive investment in its domestic medical tourism industry. The rationale for this ongoing support was reflected in an official 2014 brief titled “Medical Globalization, the Benefits Go to Citizens”, where the MOHW argued the following:

“The benefits of medical globalization go back to all people. From 2009, 630,000 foreign patients visited Korea for five years. As a result, 1 trillion won ($ 0.83 billion) of medical revenues came from this, which is equivalent to exporting 95,000 small cars. By 2017, attracting 1.5 million overseas patients will result in medical revenues of 3.6 trillion won ($2.97 billion) and will increase the number of jobs of 28,000 (as of 2017)”.

Though continuing to receive national support, policy to specifically address challenges associated with the potential negative impacts of South Korea’s medical tourism industry (such as the internal brain drain of healthcare workers and privatization of the public healthcare sector) have received less attention in the literature and in formal government documents. For example, analysis under the Comprehensive Plan concluded that since the ratio of foreign patients (1% in 2014 and projected to increase to 3% in 2020) was relatively low, the South Korean medical infrastructure would be able to accommodate the increasing number of foreign patients without having a negative impact on domestic healthcare capacity. However, this calculation did not factor in more optimistic projections of rapid growth of foreign patients hoped for to calculate this same ratio [38].

Hence, analysis of the Comprehensive Plan finds that it primarily lists positive economic benefits of medical tourism yet lacks an in-depth assessment of how the sector will affect public healthcare capacity should foreign patients increase in the numbers needed to generate significant national economic benefit. In addition, the Comprehensive Plan as well as the Act does not mention how revenue generated from hospitals or attraction agencies due to deregulation, would be used to support the public health sector should any negative impacts occur due to growth that would need to be offset [51]. Also, it is unclear whether South Korea can achieve desired results in the face of strong competition from existing regional medical tourism rivals, which could push the South Korean government to policies favoring more deregulation and increased government stimulus in order to remain competitive.

### Comparison of medical tourism policies of other top Asian destination countries

In order to better understand the policy rationale of South Korea in its continued promotion of its domestic medical tourism industry, it is important to assess the policies of other top Asian medical tourism destinations. A list of the 10 top medical tourism destinations around the world includes the following countries: India, Brazil, Malaysia, Thailand, Turkey, Mexico, Costa Rica, Taiwan, South Korea and Singapore [52]. Among these countries, a number of these “key destinations” are countries in Asia that have instituted aggressive policy initiatives seeking to promote medical tourism as a critical part of their national economic development strategy (see Table 6 for summary). Specifically, the Thai, Indian, and Singaporean governments have provided official government support for their medical tourism industries as part of a broader agenda of economic development and tourism expansion by way of various policy initiatives, including taking part in large international trade conferences and actively advertising the attractiveness of their respective medical tourism services [24, 29, 32].

**Table 6:** Comparison of Top Destination Asian Country Medical Tourism Industries

Country	Summary of Strategy	Size (foreign patients)	% of Tourism to GDP (2019)
**Thailand**	Visa procedure simplification Tax exemptions for health facilities for medical tourists Center of Excellent Health Care of Asia Initiative	2.5 million	21.6%
**India**	Lower import duties Prime land at discounted prices Tax concessions	0.85 million	9.2
**Singapore**	SingaporeMedicine (Multi agency partnership) Agreements with Middle Eastern nations	1.2 million	10.00%
**South Korea**	Medical Tourist Visa Medical institution registration system Allowing physicians and dentists to dispense medicines Medical tourism hotel business Malpractice insurance policy for a medical institution Multilanguage advertisement	0.30 million	2.7%

Sources: Strategy: Noree et al (2016), Lunt et al (2011)*,* Size: Korea Tourism Organization (2016)*,* % of Tourism to GDP: World Travel & Tourism Council (2019)

For example, the success of the Thai medical tourism private sector, such as Bumrungrad Hospital, has encouraged the government to increase government involvement [30, 40, 53]. The Thai government started the policy initiative of “Thailand: Centre of Excellent Health Care of Asia” in 2003 with the aim of attracting 850,000 new foreign patients [54]. The Bumrungrad Hospital claims to be one of the largest private hospitals in Southeast Asia (with 580 beds and more than 30 specialty centers treating 1.1 million patients annually, including over 520,000 foreign patients) and also reported US$477 million turnover (revenue) in 2013 [41]. Importantly, countries like Thailand rely heavily on traditional forms of tourism and recognize the strong economic potential of the related medical tourism industry to contribute to overall economic growth. According to the World Travel & Tourism Council, the direct contribution of travel and tourism (THB 3538.78 bn) was 21.6% of total Thailand GDP in 2018. To continue to support and promote the Thai medical tourism industry’s growth, several policy initiatives have been implemented by the government including simplification of visa procedures in order to facilitate easy entry of foreign patients, tax incentives for investment in health care facilities for medical tourists, and efforts to attract foreign patients through international advertising [14, 40].

This is similar to the growth and support strategies undertaken by India. The country implemented a new visa category - an M (medical) visa - to attract a growing number of medical tourists through streamlining the convenience of the immigration entry process [15]. In addition to immigration policy changes, it also instituted tax breaks for medical tourism providers by lowering import duties and increasing the rate of accounting depreciation for some medical equipment while also providing land at subsidized prices for hospitals providing medical tourism services [42]. Another country heavily investing in medical tourism is Singapore, which in 2003, established “Singapore Medicine”, a multi-agencygovernment-industry partnership designed to make Singapore an international medical hub [40]. The Singaporean government helped their domestic hospitals to secure international accreditation that can help attract foreign patients as it provides an additional measure of healthcare quality and regulatory oversight [15]. Singapore has also signed agreements with Middle Eastern countries and has actively promoted information about its hospitals, competitive medical costs, and aligned tourism opportunities in order to attract prospective medical tourists to the country [55].

## Discussion

Based on the results of this study, we identified 14 articles through a systematic literature review from PubMed and JSTOR related to South Korea medical tourism policy. Among these studies we found that existing literature primarily focused on the legislative history and rationale for South Korea’s enactment of pro-medical tourism policy, economic considerations and opportunities associated with growth of the industry, and the specific experiences of medical tourists visiting South Korea. There was an overall lack of studies, analytical or commentary-based, that conducted an in-depth analysis of the healthcare impact of these policies or comparing their benefits or costs to other medical tourism destinations. This is likely reflected by the fact that the majority of literature we reviewed did not address the topic from a public health or health system perspective, but instead focused on barriers to development, economic impact, effectiveness of promotional policies, and experiences and satisfaction of medical tourists. This asymmetry in the policy research agenda for South Korea is also reflected in broader published reviews of the medical tourism industry by *Virani* et al. that suggested only a small proportion of medical tourism research focuses on policy issues [56, 57]. Crucially, this could create knowledge gaps in how we understand the pros and cons of this industry and further limit the ability to generate evidence-based policymaking in favor of public health-centered outcomes.

Though a number of countries, including South Korea, have made significant economic and policy commitments to grow their medical tourism industries with the aim of promoting their domestic healthcare service and tourism sectors, the benefits remain unclear, particularly in the context of the possible negative impacts of promoting this form of domestic and international healthcare policy [58, 59]. In fact, some key risks and negative consequences have been highlighted in national debate about the Act. Specifically, during the review of the Act in the Health, Welfare, and Family Committee meeting of the National Assembly of Korea, a lawmaker argued as follows: first, a domestic patient would have less access to a tertiary hospital; second, hospitals might prioritize and provide better service to patients who bring more benefit to the hospital by paying with non-national insurance funds (i.e., foreign patients paying out of pocket), which could preclude medical services to domestic patients with national insurance; and third, medical tourism could represent a slippery slope to privatization of public medical institutions.

Beginning with the first concern of limiting access for domestic patients, internal “brain drain” represents a key concern with medical tourism, including in South Korea. This occurs when healthcare providers in destination countries are “captured” by the medical tourist patient population by instead serving the medical tourist market at the expense of domestic patients. The creation of a higher-paying privatized market with insufficient regulation can cause healthcare providers to shift from treating patients in the lower-paying public health sector. For example, *Noree* et al. concluded that the Thai health system has suffered from a shortage and inequitable distribution of physicians due to shifts from public to private hospitals and that increased numbers of medical tourists are worsening this situation [38]. India, another top medical tourism destination has also suffered from shortages of healthcare professionals in public settings, with the private sector now emerging as the main employer of the healthcare workforce [59]. Hence, an influx of business activity for medical tourism can heavily skew the distribution and availability of the domestic healthcare workforce if internal ‘brain drain’ is not properly managed [60].

Related to the second concern raised during the Act’s debate, the internal brain drain, which impacts a country’s doctor-to-patient ratio in the public health sector can also lead to a “two-tiered” healthcare system. This occurs when foreign and certain domestic patients benefit from access to better private healthcare services with more favorable doctor-to-patient ratios and access to state-of-the-art medical facilities, while local, rural and poorer populations may be limited to lower quality care or resource strained healthcare access [17]. This distorts healthcare expenditures and can lead to ‘cream skimming’, where rich patients with better access and ability to pay gain greater access to healthcare services at the expense of patients who already lacked access, potentially worsening existing health disparities [60].

Domestic promotion of medical tourism can also lead destination country governments to shift already scarce public healthcare resources away from essential services and benefits towards more resource-intensive activities in order to compete for the business of foreign patients, given that the medical tourism industry primarily competes on quality and price [38, 41, 54]. Destination countries are also more likely to invest in their medical tourism industries through direct funding, tax subsidies, and land grants, all incentives that can crowd-out public investments allocated towards critical public health infrastructure and programs. This can consequently lead to greater privatization at the expense of public institutions, the third concern raised.

Although national governments are expected to play a critical role in regulating the private healthcare sector (including the medical tourism industry), state interventions or remedial actions by the South Korean government that have been implemented thus far appear to be minimal. Critically, even though concerns have been raised and South Korean policymakers purposefully shaped the Act based on strategies of competitors, policy learning has been selective, with many of the remedial mechanisms used by other countries to guard against the problematic aspects of their own domestic medical tourism industries neglected [56, 57]. For example, Singapore has attempted to address the issue of internal brain drain by recruiting high quality healthcare workers from other neighboring countries and offering competitive salaries, training and career development opportunities [56]. Singapore has also reversed its policy position on promoting public sector participation in medical tourism, shifting to restrict public hospitals from marketing to foreign patients to address shortages of hospital beds [56].

Further, profits gained from the medical tourism industry are not likely to “trickle down” to other public programs, one of the benefits argued by proponents and supporters of medical tourism. This “redistribution” is inherently difficult given a non-redistributive tax system, a largely foreign-owned medical tourism sector, and the potential negative impacts of deregulation or possible presence of corruption. Therefore, although a destination country gains economically from medical tourism (for example, in terms of increased GDP), it does not necessarily equate to gains for the overall healthcare system due to resulting healthcare workforce shortages, negative impact on availability of specialty care areas, and skewed tax benefits [38]. In response to many of these challenges, Thailand has removed restrictions on recruiting international medical graduates, levied a tax on medical tourism services to fund its health systems, reviewed policies on long-stay tourism, and restricted tourists from using certain specialties [56]. These remedial responses by other countries are critical policy lessons and tools that the South Korean government should consider in future medical tourism policymaking.

Given South Koreas’ heavy reliance on the private sector for healthcare service provisioning, the possibility of a two-tiered system emerging that disproportionately prioritizes resource allocation for medical tourism patients compared to the general public should be recognized as a critical concern in future policymaking [31]. In response, the South Korean government should carefully weigh the pros and cons of continuing to aggressively promote its medical tourism industry, including addressing concerns that political and competitive pressures will lead to increasing calls for privatization. Hence, assessing the tradeoffs between economic gains and the larger health policy impact of medical tourism on the entire South Korean health system and its citizen’s health is needed. This is particularly important in countries like South Korea, which have committed to Universal Health Coverage (UHC).

### What lies ahead for the future of the Korean medical tourism industry?

In 1989, the Republic of Korea (e.g. South Korea) achieved UHC, resulting in the entire population gaining access to either the national health insurance scheme (about 97% of the population) or from the tax-based Medical Aid Program (about 3% of the population) [31]. In contrast to public health financing through national health insurance, healthcare delivery is quite dependent on the private sector despite some public health facilities available for poorer and disadvantaged populations at the local level [61]. As an example, in 2012, the private sector accounted for approximately 94% of all hospitals and 88% of total beds [61]. Although the number of healthcare professionals has continuously increased in response to growing demand for healthcare services resulting from expanding health insurance coverage under UHC, the number of South Korean healthcare professionals is still below the average of other OECD countries [61].

Behind this backdrop, proponents of medical tourism have evaluated the South Korean government’s efforts to promote medical tourism and found it insufficient compared to efforts of rival countries even following passage of the Act [62]. A 2013 report by the Korea Institute for Industrial Economics & Trade (KIET) reported that ongoing weaknesses in the South Korean medical tourism sector were caused by low awareness and lack of sufficient public relations, inadequate infrastructure to support medical tourism services, lack of integrated support systems, overregulation that hinders activation of medical tourism, and intensifying competition from competitors [62]. Based on this evaluation, the report recommended several additional policy actions to secure South Korea’s competitive advantage in the global medical tourism industry with a focus on implementing extensive deregulation by: (a) improving investment conditions by allowing establishment of open investment (similar to for-profit) medical corporations; (b) allowing remote medical services between physicians and patients; (c) expanding the number of beds for foreign patients in hospitals; and (d) attracting large numbers of foreign patients through insurance companies [62]. However, the report did not highlight the potential consequences of deregulation of the sector, including concerns about maintaining quality of services, potential lack of oversight of private medical corporations and their intermediaries (e.g. including brokers, online sites and other intermediaries), and further unregulated privatization of health markets [63].

Some of the recommendations in the KIET report, such as allowing for remote medical services and the expansion of the number of beds for foreign patients, were introduced in both the Act and other government policy documents. The report by Korean Institute of Hospital Management (KIHM) also requested that the government change the limit on the number of beds for foreign patients in tertiary hospitals by increasing the ratio from 5 to 10%. The collection of these policy recommendations by proponents in favor of increasing investment in medical tourism heavily focuses on deregulation and increasing access for foreign patients to South Korean health services despite potential negative impacts for domestic patient access. Hence, it is important to reconcile the distortionary effects that these investments will have on local health systems and the communities they serve, along with the need to counteract them with remedial policy mechanisms other countries have implemented [56]. Otherwise, the health needs and wellbeing of South Korea’s own citizens will likely be compromised along with national goals of maintaining UHC [56].

Regional competitive market forces are also at play, with strong competition from existing Asian country competitors. KIET analyzed Japan and China’s increasing efforts to promote medical tourism, such as China’s designation of the Shanghai International Medical Zone and Japan’s national support of medical tourism as a key future industrial sector, as major emerging threats [62]. New competition from Taiwan is also attracting patients from Mainland China and the anti-Korean cultural movement among far-right groups in Japan has also discouraged Japanese patients from traveling to South Korea for medical treatment though the countries are close in geographic proximity [64]. More fundamentally, although the current cost of medical treatment in South Korea is internationally competitive, it is higher on average than that of Thailand and India, mainly due to higher labor costs [64, 65].

Hence, despite the South Korean government’s effort to promote medical tourism for the past decade, its performance appears to fall behind other major medical tourism destinations in the region. Other countries have addressed regional competitive challenges with market specialization. For example, Singapore has taken advantage of its technological and logistical capabilities (including government investment in innovation, a globally-oriented medical education system, accreditations systems based on international standards, and technology infrastructure) and managed to create a unique niche in the global medical tourism market for its services [56], which could serve as a potential roadmap for future strategic investment and specialization by South Korea. Critically, overreaction by acquiescing to strong demands to relax regulations and expand government support through revision of the Act from proponents of further medical tourism expansion, such as hospitals and insurance companies, needs to be carefully critiqued.

### Limitations

Our literature search was limited to the academic databases PubMed and JSTOR and the grey literature search was limited to search results prioritized by the Google search engine. PubMed and JSTOR were chosen based on relevance to the medical and life sciences literature (PubMed articles) and social science and policy-related articles not specific to medical or healthcare literature (e.g. JSTOR). Articles not indexed in PubMed and JSTOR, as well as the results beyond the first 50 search results from Google search were not included in this study which could have impacted study results. Further, we limited our structured keyword searches using Boolean searches on PubMed and JSTOR to the title and abstract of articles, which may have limited the number of articles returned for review. Lack of standardized approaches across selected studies presented challenges in assessing the potential influence of bias on the cumulative evidence for this body of literature. Also, we made subjective assessment of inclusion of articles based on relevance to South Korean medical tourism policy per our inclusion and exclusion criteria, which could have impacted results, but we also note that when we conducted keyword searches on general text of each article, very few directly addressed or were relevant to South Korea’s medical tourism policy.

## Conclusion

Similar to other top destination countries for medical tourism, the collection of supporting policies spanning the Act, the Comprehensive Plan, and the Enforcement Plan enacted by the South Korean government has brought some economic benefit to the country and may similarly motivate other countries to pursue investment in this sector. However, lessons discussed in this review from the South Korean policy process, as well as other top destination countries, including Mexico, India, Thailand, and Malaysia, where policymaking is mostly focused on developing supply-side capacities and increasing competitiveness, often to the detriment of local and community health needs in absence of regulatory controls [56], should be acted upon with caution. Inherently there remains an underlining tension between medical tourism policy goals of economic growth and the need to maintain resilience and equity in domestic public health systems. Further, the need to establish international quality standards for international medical treatment, harmonizing legal, trade, migration and economic frameworks associated with multinational tourism services, and ensuring that patient choice is optimal for both domestic and tourism-seeking populations, continue to be critical concerns that need to be addressed by the broader global medical tourism industry [66–68].

Following the impeachment of former South Korean President Park Geun-hye and election in 2017 of opposition party candidate and current President Moon Jae-in, past policy positions that have continuously promoted medical tourism over the past decade need rethinking. The Moon administration should leverage its mandate for change and reform, including plans to reform and expand the country’s healthcare system, by looking carefully into the potential benefits and perils of continuing on the pathway of medical tourism expansion [69]. Critical in this approach should be an honest assessment of how medical tourism not only contributes to national economic output, but also how it can negatively impact domestic healthcare costs, access and affordability to treatment, and South Korea’s commitment to UHC.

## Data Availability

Data sharing is not applicable to this article as no datasets were generated or analysed during the current study.

## Abbreviations

GDP: Gross domestic product
HIV: Human immunodeficiency viruses
IMC: International Meditour Coordinator
KFHR: Korean Federation of Medical Activists Groups for Health Rights
KHIDI: Korea Health Industry Development Institute
KIET: Korea Institute for Industrial Economics & Trade
KIHM: Korean Institute of Hospital Management
KOSIS: Korean Statistical Information Service
MOHW: Ministry of Health and Welfare
OECD: Organisation for Economic Co-operation and Development
UHC: Universal Health Coverage
